# The Secret of Institutional Discipline

**Published:** 1914-05-23

**Authors:** 


### THE SECRET OF INSTITUTIONAL DISCIPLINE.

Very rarely, considering the number of human beings collected in them, do grave troubles or disturbances occur in institutional life, and therefore we may regard the institutional worker as peculiarly fitted to give a few practical hints on such problems as that with which the recently formed organisation, known as the Duty and Discipline Movement, desires to deal. The first-fruit of this movement, from a public point of view, is the issue of a small volume of essays reprinted from various periodicals, and gathered together under the general title of "Anarchy or Order?" On reading the opening paper, by Helen Colvill, on "Anarchy," we realise to what length the squeamishness of middle age can go, and wonder why any writer with still enough good sense left to describe the remark of hers to a friend—"You and I see the beginning of the end; our children will be in at the death"—as "silly," yielded to the temptation to spin it out to an essay of some sixteen pages, and, what is worse, to reprint it here. In the faint but inextinguishable hope of finding a redeeming feature in a volume with a pretentious title which made such a blunder at its start, we read to the end, and wish to deal with two of the most forcible essays in the volume, entitled respectively, "Experience Teaches," by Mr. A. Maynard, and "How to Deal with Obstinate Children," by Mr. G. W. Hart. Taking the latter first, any unbiassed person reading it, and noting the relish with which italics are used, will be forced to the unpleasant conclusion that the Duty and Discipline Movement is nothing but a crusade to revive child flogging on the largest possible scale. That is certainly the heart and kernel of Mr. Hart's advice on the subject he has chosen to discuss. We really have very little patience with a clergyman, for so Mr. Hart's prefix shows him to be, so ill-considered a student of the Bible, and so oblivious to the plain meaning of words, as to imagine that Solomon's advice, "spare the rod and spoil the child," which he misquotes with really unpleasant unction, gives Biblical authority for "a good, sound whipping." Solomon's son was Rehoboam, one of the most wicked and disastrous of Israel's kings, and that a clergyman of the present day should affect not to see that the story ends not with Solomon's dictum, but with the disastrous consequences of it in the person of his child, is almost too much for a layman to stomach. The moral of the story is, of course, that a clever man addicted to voluptuousness will certainly preach flogging, which is only voluptuousness in a very degraded form. Solomon was certainly a wise man, and equally certainly addicted to voluptuousness. We are, therefore, directed by the inspired writer to see that the precepts even of Solomon must be judged by their effect upon conduct, and what this effect was is painted in Holy Writ, in unmistakable colours, in the life and crimes of Rehoboam.

It is, therefore, with a sense of immense relief that we turn to the article entitled "Experience Teaches," which is the best and ablest contribution in the whole volume; and, by an irony apparently unnoticed by the editor of the collection, provides the condemnation of the two articles which we have already referred to, and of others stained by the same defect.

The truth is that Mr. A. Maynard, the author of this instructive essay, has had institutional experience himself, and thereby differs from the amateur theorists already referred to. He is quite justified in his sub-title, which reads, "An Account of a Successful Experiment in the Training of Children." It would seem that he has been in charge of an orphanage, and, as his essay shows, has learnt there by the test of practical experience the inevitable failure of the methods so rashly put forward by his fellow-contributors. Experience has taught him that discipline means not coerciveness but rather, as the derivation of the term implies, discipleship; that punishment is ruled out in practical administration because it fails to make the subjects of it realise in their souls the responsibility of their actions. He knows that the way to reach the soul is not through the skin, and insists that the "old incentive fear" is one of "the dangers which it is necessary to guard against in Institution training." After the old coercive methods had proved their failure and demoralisation on both pupils and authorities, he did not hesitate to throw them overboard and to put in practice the obvious principle, which he quotes approvingly, that "it is liberty alone that fits a man for liberty." The cases in which he gives the practical application of this principle are most instructive. Realising that "the liberty to choose the wrong way must be given while the children were still under the care of those who, whilst allowing them to suffer the consequences of their fault to the full, would stand by as friends . . . pointing out the right path for the future"; he quotes the case of a lazy girl who lost her situation by this fault. Instead of being "punished," passed on, with a false character, or allowed to return to the school, she was boarded out, and unable to secure a situation through her bad "character" was left for six weeks in a solitary condition. At the end of this time she realised that the penalty of laziness, that of being without employment, was worse than the fatigue of even hard work, begged to be taken back, and remained twenty months in a place thereupon secured for her, where we are led to believe she is still. In short, responsibility was brought home to her in the only permanent way that it can be brought home to anyone—by experiencing some results of acting foolishly. No one ought to be able to harm a person except himself or herself, and this truth, which is the basis of responsibility, and therefore of discipline, as distinguished from coercion, supplies the basic argument against punishment in all its forms. Punishment is condemned because, being inflicted from without by another, the subject of it feels he or she has contracted out of his or her offence and "paid for it" by undergoing castigation. Most active natures are quite willing to offend again on these terms, and that is how the habitual criminal is manufactured. Institutional officers, who have no punishment at command except dismissal, have known this all along; but they and the rank and file of the Duty and Discipline Movement may tender sincere thanks to Mr. Maynard for his unimpeachable testimony to the true secret of institutional discipline.

